# Factors Affecting the Color Change of Monolithic Zirconia Ceramics: A Narrative Review

**DOI:** 10.3390/jfb16020058

**Published:** 2025-02-11

**Authors:** Ebru Binici Aygün, Esra Kaynak Öztürk, Ayşe Bilge Tülü, Bilge Turhan Bal, Seçil Karakoca Nemli, Merve Bankoğlu Güngör

**Affiliations:** Department of Prosthodontics, Faculty of Dentistry, Gazi University, Ankara 06490, Türkiye; ebru-binici@hotmail.com (E.B.A.); kynkesra03@gmail.com (E.K.Ö.); bilgetulu1998@gmail.com (A.B.T.); bilgeturhan@gmail.com (B.T.B.); secilkarakoca@yahoo.com (S.K.N.)

**Keywords:** monolithic zirconia, color, translucency

## Abstract

Zirconia restorations are widely used in dentistry due to their high esthetic expectations and physical durability. However, zirconia’s opaque white color can compromise esthetics. Therefore, zirconia is often veneered with porcelain, but fractures may occur in the veneer layer. Monolithic zirconia restorations, which do not require porcelain veneering and offer higher translucency, have been developed to address this issue. Zirconia exists in three main crystal phases: monoclinic, tetragonal, and cubic. Metal oxides such as yttrium are added to stabilize the tetragonal phase at room temperature. 3Y-TZP contains 3 mol% yttrium and provides high mechanical strength but has poor optical properties. Recently, 4Y-PSZ and 5Y-PSZ ceramics, which offer better optical properties but lower mechanical strength, have been introduced. This review examines the factors affecting the color change in monolithic zirconia ceramics. These factors are categorized into six main groups: cement type and color, restoration thickness, substrate color, sintering, aging, and zirconia type. Cement type and color are crucial in determining the final shade, especially in thin restorations. Increased restoration thickness reduces the influence of the substrate color while the sintering temperature and process improve optical properties. These findings emphasize the importance of material selection and application processes in ensuring esthetic harmony in zirconia restorations. This review aims to bridge gaps in the literature by providing valuable insights that guide clinicians in selecting and applying zirconia materials to meet both esthetic and functional requirements in restorative dentistry.

## 1. Introduction

Zirconia is favored for dental restorations due to its superior esthetics compared to metal-ceramic restorations and its excellent mechanical properties, enhanced by transformation toughening. Additionally, advancements in chairside milling and rapid-sintering technology have made the fabrication process more automated, precise, and time-efficient [1]. Although the opaque white color of zirconia, resulting from its polycrystalline microstructure, negatively impacts esthetic appearance; the level of opacity can vary among the different zirconia systems available on the market [2,3]. For this reason, zirconia is commonly used as a core material in dental restorations and is veneered with porcelain to enhance esthetics [3]. However, veneering the zirconia core with ceramic increases the overall thickness of the restoration, necessitating greater tooth reduction and potentially weakening the abutment tooth. Mechanical issues such as chipping and fracture are also common in veneering porcelain. To overcome these challenges, monolithic zirconia was introduced as an alternative. Monolithic zirconia now allows for more translucent and esthetic restorations [1].

Zirconia (ZrO_2_) exists in three primary crystalline phases; monoclinic at room temperature, tetragonal above 1170 °C, and cubic at 2370 °C [4]. As zirconia ceramics cool, they undergo a transformation from the cubic to the monoclinic phase, leading to a volumetric expansion of around 5%. This transformation can cause fractures and make zirconia more brittle. As a result, pure zirconia is not used in biomedical, structural, or functional applications due to challenges associated with its phase transformation and volumetric expansion [5]. Metal oxides such as magnesium, cerium, yttrium, and calcium are used to stabilize the tetragonal phase of zirconia at room temperature [5,6]. In dentistry, yttrium oxide (Y_2_O_3_) is the most commonly used oxide for stabilizing zirconia [7]. When the added Y_2_O_3_ content is above 8 mol%, the material remains in a fully stabilized cubic phase [8]. With the addition of smaller amounts (3 mol% to 5 mol%), yttria partially stabilized zirconia (Y-PSZ) is formed [5]. When a fracture occurs in the Y-TZP material, its grains absorb energy, triggering a transformation of the crystals from the tetragonal phase to the monoclinic phase. The zirconium grains undergo a 4% volume increase during the tetragonal–monoclinic phase transformation. As a result, the localized compressive stress fields prevent further fracture propagation. This process is known as transformation toughening. The transformation toughening of zirconia enhances its physical properties, which provides high flexural strength and fracture toughness, making it suitable for demanding applications [7]. Although monolithic Y-TZP ceramics have recently gained popularity due to their good biocompatibility, esthetic properties, and high strength, they still present significant drawbacks, including semi-translucency, radiopacity, and limited light transmission [9,10].

The first dental application containing 0.25–0.5 wt.% alumina is traditional zirconia, known as 3Y-TZP [8]. 3Y-TZP is partially stable in the tetragonal phase, exhibits high mechanical properties, and was introduced more than 25 years ago. Due to its biocompatibility and flexural strength exceeding 1000 MPa, 3Y-TZP is commonly used as a core material [11]. Its mechanical properties also allow it to be used in multi-unit restorations. However, its high opacity limits its optical properties, negatively impacting its esthetic appearance. In 2013, enhanced versions of 3Y-TZP materials were introduced to the market. This improvement was achieved through high-temperature sintering to minimize porosity and significantly reduce the alumina content [1]. By decreasing the alumina content to less than 0.05%, highly translucent 3Y-TZP has been developed, significantly enhancing its translucency [8]. Next, further improvements in zirconia translucency were achieved by increasing the yttria (Y_2_O_3_) content to 5 mol% (5Y-PSZ), resulting in approximately 50% cubic phase content [12]. The translucency of zirconia was further enhanced in this material, making it possible to use monolithic zirconia restorations in anterior teeth [8]. However, a higher cubic phase content adversely affects mechanical properties, including flexural strength and fracture toughness.

**Figure 1:**
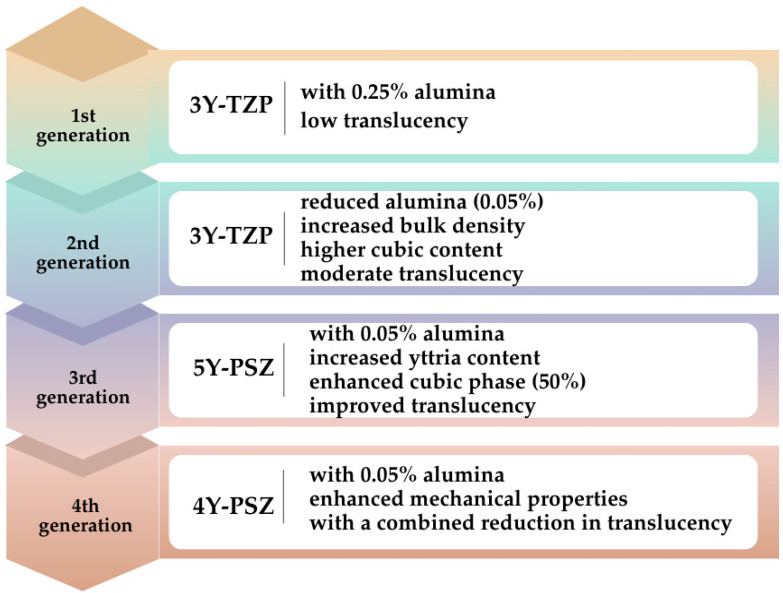
The contents and translucency levels of the generations of the zirconia ceramics.

Considerable effort has been dedicated to optimizing the material properties through various modifications to expand the range of indications for monolithic zirconia restorations. In 2017, 4Y-PSZ was introduced to the market. Compared to 5Y-PSZ, the yttria content in 4Y-PSZ has been reduced to 4 mol%, leading to improved mechanical properties while compromising optical characteristics. According to the manufacturer, 4Y-PSZ restorations are suitable for anterior and posterior crowns, as well as short-span fixed partial dentures with multiple units [12,13]. In contrast, 5Y-PSZ restorations are primarily indicated for anterior crowns and short-span fixed partial dentures [13]. In general, increasing the yttria content enhances the material’s optical properties but compromises its mechanical strength [14]. Among yttria-stabilized zirconia materials, 3Y-TZP offers the highest mechanical strength, yet its high opacity restricts its application to non-esthetic areas in dentistry. In contrast, 5Y-PSZ offers superior optical properties, although its mechanical strength is reduced. 4Y-PSZ is considered an intermediate material, balancing optical and mechanical performance. Despite its lower strength, translucent 5Y-PSZ is a suitable option for anterior restorations, as it meets the minimum strength requirements while providing a clinically acceptable esthetic outcome [15]. Increasing the proportion of the cubic phase in the zirconia structure enhances the material’s translucency. The cubic phase ratio has progressively increased throughout the generations of zirconia. However, studies indicate that a higher cubic phase content reduces flexural strength, fracture toughness, and fatigue resistance [16]. A higher yttria content leads to an increased cubic phase and greater translucency. However, a reduction in the tetragonal phase, which contributes to flexural strength, results in decreased fracture toughness. Therefore, the translucency of 5Y-PSZ is approximately 20% to 25% higher than that of 3Y-TZP, whereas its flexural strength is from 40% to 50% lower [8]. In summary, while third-generation zirconia exhibits superior optical properties, fourth-generation zirconia provides greater durability [17]. The contents and translucency levels of the zirconia materials in the generations are summarized in Figure 1.

Recently, polychromatic or color-gradient zirconia blocks have been introduced to replicate the natural color transitions of teeth [14]. Some zirconia materials integrate different generations with varying yttria content within a single block, combining high strength and enhanced translucency [12]. This typically involves high-strength zirconia in the dentin or body region and high-translucency zirconia in the incisal or occlusal areas to improve esthetics [18]. Due to these advancements, dental zirconia is classified into three types: monochromatic zirconia ceramics with uniform composition, polychromatic multilayered zirconia ceramics, and polychromatic hybrid-structured multilayered zirconia ceramics [19].

In dentistry, color is considered a measurable quantity rather than a subjective quality. Therefore, color-determining systems and color parameters are used to evaluate the optical properties of the restorations. The Munsell Color System defines the three dimensions of color as hue, value, and chroma, and the CIELab Color System defines color along three axes as L*, a*, and b*, among the most commonly used systems [20]. ΔE (delta E) is a unit used to quantify color differences between two shades. This term is mainly used in dentistry and other esthetic-related disciplines to evaluate the color compatibility of restorations with natural teeth. The calculation of ΔE is performed using a color system [20,21]. In the CIELab system, formulas such as ΔE_ab_ and ΔE_00_ are used to determine color differences. Although the ΔE_00_ formula provides results that better align with the human eye’s perception of color differences, ΔE_ab_ is more commonly used in dentistry due to its simplicity [20]. The color of the restoration is compared with an adjacent tooth or target shade to calculate the ΔE value, which is then evaluated based on the perceptibility (ΔE_ab_ = 1.2) and acceptability (ΔE_ab_ = 2.7) thresholds [22]. However, ΔE_00_ defines the perceptibility and acceptability threshold values as 0.8 and 1.8, respectively [22]. The dentist must select the most suitable color for the patient and transfer it correctly to the laboratory. Color selection is performed using different methods divided into visual and instrument-based color selection [23]. In visual color selection, standard color scales are used. The first systematic color scale was the “Tooth Color Indicator”, developed by Clark, which contained sixty ceramic specimens. Although many products were introduced afterward, the real breakthrough occurred with the launch of the “Vitapan Classical” (VC; Vita Zahnfabrik, Bad Sackingen, Germany) color scale in the mid-1950s. The subsequent development came in the late 1990s with the creation of the “Toothguide 3D-Master” (TG; Vita Zahnfabrik, Bad Sackingen, Germany). Finally, the “VITA Linear Guide 3D Master” (Vita Zahnfabrik; Bad Sackingen, Germany) color scale was introduced [24]. Visual color selection is inherently subjective and may vary from person to person. It can also be influenced by the observer’s gender, age, cultural background, eye fatigue, and environmental factors. However, visual color selection continues to be the predominant method in dentistry. In contrast, instrument-based color selection methods have been developed to achieve greater esthetic accuracy and reliability. These methods use spectrophotometers, colorimeters, and digital cameras [25]. An effective color-measuring tool must meet several criteria, including shock resistance, ease of operation, rapid measurement capability, durability, having a suitable light source, affordability, and most importantly, providing accurate and precise results. Digital methods minimize subjectivity, and digital photography, in particular, has transformed dentistry. It facilitates the documentation of patients’ oral conditions, comparison of treatment progress, shade selection assistance, and patient education. Digital single-lens reflex (DSLR) cameras, equipped with adjustable lenses and various settings (aperture, ISO, and shutter speed), have become essential tools in modern dental practice. While reliable lighting and fixed distances are crucial, digital photography ensures accurate shade matching when conducted under controlled conditions [26].

Current ceramic materials are suitable for optimal esthetics; however, there is an increasing demand for more durable materials because of their brittleness. Highly translucent zirconia-based ceramics, manufactured through computer-aided design and computer-aided manufacturing (CAD-CAM) technology, are becoming increasingly popular due to their excellent physical, mechanical, biological, and chemical properties. However, various factors can influence the final color of zirconia restorations, including the type and color of cement, substrate color, restoration thickness, sintering, aging, and the type of zirconia material [27,28,29,30,31]. These parameters should be carefully evaluated before treatment to achieve successful esthetic outcomes in zirconia restorations. Thus, this narrative literature review aims to discuss these practical factors affecting the color change in monolithic zirconia restorations based on the literature. The methodology for developing this article is as follows: Relevant studies were identified through searches in Google Scholar, PubMed/Medline, and Web of Science databases, focusing on publications between 2008 and 2024. The search was conducted using search terms such as ‘monolithic zirconia’, ‘color’, and ‘color change’. Articles in English were included. This study focused on narrative reviews, including systematic reviews, reviews, and in vitro studies addressing factors affecting the color change in zirconia restorations. However, letters to the editor and case reports were excluded from the review. Articles from all categories of journals were included, provided they met the specified inclusion and exclusion criteria.

## 2. Factors Affecting the Color Change in Monolithic Zirconia Ceramics

### 2.1. Effects of Cement Type and Cement Color on the Color Change in Monolithic Zirconia Ceramics

The cementation of zirconia restorations can be performed using traditional cements or adhesive cementation, either with or without additional adhesive systems. Resin cements are the most commonly used dental materials for cementing zirconia restorations because they offer good esthetics, low solubility, high strength, and mechanical resistance [32]. However, zirconia has a polycrystalline tetragonal structure. Lacking a glass phase, zirconia is unsuitable for silanization and resistant to etching with acids such as hydrofluoric or orthophosphoric acid. Therefore, alternative protocols have been developed to improve bonding strength [33]. Various mechanical and chemical surface treatments enhance the bond strength between zirconia and resin cement. Mechanical treatments include airborne particle abrasion, tribochemical silica coating, selective infiltration etching, and laser treatment [34]. Chemical surface treatments include silanization, hydrofluoric acid etching, hot etching, and the application of primers containing 10-methacryloyloxydecyl dihydrogen phosphate. In addition, combining chemical and mechanical techniques is recommended for an effective result [35]. Minor alterations in the microstructure or surface roughness from internal surface finishing can affect color and transparency due to the material’s thinness and high translucency, particularly when using ultra-transparent zirconia in minimally invasive restorations [36]. Chen et al. [36] evaluated the effects of different surface treatments and thicknesses on ultra-transparent zirconia’s color, transparency, and surface roughness. One-hundred-and-twenty ultra-translucent zirconia specimens were divided into groups based on thickness (0.3, 0.5, and 0.7 mm) and surface treatments (control, airborne particle abrasion, lithium disilicate coating, and glazing). Results revealed that surface treatments and thickness significantly influenced color difference (ΔE_00_), translucency, and surface roughness. Airborne particle abrasion resulted in the lowest transparency, highest color difference, and excellent surface roughness. Thinner zirconia (0.3 mm) showed a strong negative correlation between surface roughness and translucency, while increasing thickness reduced the impact of surface treatments on optical properties. From a clinical perspective, the findings highlight the importance of balancing bonding enhancements from surface treatments with their effects on the optical properties of ultra-transparent zirconia and suggest that increasing restoration thickness can mitigate adverse impacts on esthetics.

Furthermore, the cement type and color affect the restoration’s final color [37,38]. The effects of cement on the optical properties of restorations have become even more critical, especially with the advances in highly translucent zirconia materials that can be used in the anterior region [37]. Therefore, the type and color of the cement used to lute zirconia restorations should be carefully selected. Studies on the effects of cement type and color on the color change in monolithic zirconia ceramics are summarized in Table 1.

These studies have shown that the type and color of the cement used in zirconia restorations play a crucial role in determining the final esthetic outcome. The recent introduction of 5Y-PSZ and 4Y-PSZ restorations, which offer high translucency, makes them particularly suitable for use in the anterior region. These restorations can reflect the color of the underlying tooth or abutment, thus enhancing the overall natural appearance of the restoration. However, the cement type and color become critical, especially when using highly translucent materials over substrates with different colors. For instance, when the abutment tooth or the substrate has a discolored or dark hue, the cement’s opacity becomes increasingly essential. In such cases, more opaque cements are recommended to prevent undesirable colors from reflecting the underlying structure, which can affect the final esthetic result. Therefore, it is essential to carefully consider both the tooth color and the zirconia material’s optical properties before determining the appropriate cement color and type. Additionally, the interaction between the restoration material and the cement should not be underestimated. Certain cements may alter the appearance of the zirconia, potentially leading to color mismatches. For optimal esthetic results, selecting cements that match the zirconia’s translucency or provide a slight contrast, depending on the desired effect, is crucial. This careful selection ensures the final restoration meets functional requirements and achieves a highly natural and harmonious appearance in the patient’s smile.

### 2.2. Effect of Restoration Thickness on the Color Change in Monolithic Zirconia Ceramics

It was reported that material thickness affected the optical properties of the material [44]. The thicker the material, the longer the path light takes through the material. As a result, more light is absorbed and diffused, and the amount of light passing through the material decreases. This causes the material to have different optical properties at various thicknesses. On the other hand, increasing the thickness of a zirconia restoration can improve its masking properties by reducing translucency [31]. Before treatment, it is necessary to determine the ideal restoration thickness under the existing conditions. Studies on the effect of zirconia thickness on the color change in the final restoration are summarized in Table 2.

According to these studies, the final color of the restoration changes depending on the thickness of the material. New-generation zirconia ceramics generally have high translucency. However, as the thickness of zirconia restorations increases, the passage of light decreases, and the material’s ability to transmit light reduces. This helps thicker restorations become opaquer and mask the color of the underlying structure. Increasing the thickness of the zirconia restoration can be beneficial for dark-colored substrates from an esthetic perspective, as the thickness helps conceal the color of the substrate and provides a more natural appearance. However, as the thickness increases, the translucency of the restoration decreases, making it essential to maintain an esthetic balance. Therefore, the ideal thickness should be determined before treatment, ensuring a balance between esthetics and function.

### 2.3. Effect of Substrate Color on the Color Change in Monolithic Zirconia Ceramics

The color match between a dental restoration and the adjacent natural teeth depends partly on the degree of translucency of dental restorative material [49]. High translucent zirconia materials have recently been introduced to the market to provide high esthetics. These materials allow light to be transmitted and diffused into the restoration so that the tooth or the substructure’s color can significantly affect the final color [29]. Therefore, the background color should be carefully evaluated before treatment, and the appropriate restorative material should be selected accordingly. The studies on the effect of substrate color on the color change in monolithic zirconia ceramics are summarized in Table 3.

According to these studies, achieving a color match in dental restorations is crucial for obtaining an esthetically pleasing result with natural teeth. Increasing the translucency of zirconia materials is a standard method to improve color matching between the restoration and natural teeth. These high-translucency zirconia materials allow light to pass through and diffuse into the restoration, enabling the underlying material’s color to influence the final color significantly. Therefore, evaluating the substrate’s color carefully before treatment is essential. Additionally, selecting the appropriate material is key to achieving esthetic and functional success. Considering the substrate color’s effect on the restoration’s overall appearance, making an informed choice is a critical step in the treatment process. This ensures patient satisfaction and long-lasting esthetic results throughout the treatment.

### 2.4. Effect of Sinterization on the Color Change in Monolithic Zirconia Ceramics

Sinterization is a process that allows the material to be concentrated at high temperatures under the melting temperature. This process improves the mechanical properties of the material. Sinterization methods can be classified as standard, speed, and high speed [54]. It is known that temperature and holding time at this temperature, through the application of different sintering protocols, affect the physical properties of zirconia, especially of multilayered monolithic zirconia [55,56,57,58]. Studies on the effect of sinterization on the color change in monolithic zirconia ceramics are summarized in Table 4.

According to these studies, the sintering time and temperature can significantly affect the grain structure and zirconia materials’ optical properties. These parameters directly impact zirconia’s porosity and microstructure, which are key determinants of its performance. Modifying sintering parameters to increase grain size can improve translucency, making zirconia more suitable for esthetic applications. However, this comes with trade-offs: higher sintering temperatures and longer durations lead to grain growth, which can reduce the material’s mechanical strength due to phase transformations, such as the tetragonal-to-monoclinic phase transformation. Therefore, carefully optimizing sintering parameters is essential to balance translucency and strength, ensuring zirconia’s suitability for various dental and esthetic applications.

### 2.5. Effect of Aging on the Color Change in Monolithic Zirconia Ceramics

Dental materials are exposed to temperature changes, moisture, and mechanical effects in the mouth. These effects cause changes in material properties during the use process. Artificial aging is used to examine changes in vitro depending on the duration of use [65]. Monolithic zirconia undergoes LTD over time, starting at the surface and progressing inward. LTD reduces fracture load by 20–40%, increases surface roughness, causes microcracks, and weakens mechanical properties, regardless of surface treatment [7]. Several factors influence zirconia’s ability to withstand low-temperature degradation (LTD), including the yttria content, grain size, cubic phase content, concentrations of Al_2_O_3_ and SiO_3_, and the residual stress level. It was suggested that the Al_2_O_3_ content should be maintained at a minimum of 0.15 weight percent, with an optimal range of 0.15 to 0.25 weight percent, to mitigate the aging process. Lowering alumina content to enhance translucency increases the risk of susceptibility to LTD [7]. Third-generation zirconia has better-aging resistance than fourth-generation zirconia due to its higher cubic phase content [17].

The stability of the optical properties of restoration after aging is one of the main factors determining the success of the material used. After aging, the structure of zirconia may be affected, and the optical properties of the material can change. Since zirconia restorations are in direct contact with the challenges of the oral environment, such as moisture, pH changes, mechanical stresses as a source of external stress, and low temperatures critical for the stability of the tetragonal phase, different artificial aging methods have been investigated in the literature to predict the t→m phase transformation of these materials [66,67,68,69]. Ultraviolet aging caused zirconia-based restorations to become darker, yellow, redder, and more saturated, resulting in increased opacity [17]. Artificial aging processes applied to zirconia materials can generally be stored in aqueous media, hydrothermal aging, mechanical aging, hydrothermal and mechanical aging, and chemical aging. The studies on the effect of aging on the color change in monolithic zirconia ceramics are summarized in Table 5.

Zirconia restorations are exposed to various challenges in the oral environment, including temperature fluctuations from hot and cold foods, mechanical stresses from mastication, and pH variations caused by dietary acids and salivary changes. These factors can cumulatively impact the long-term performance and color stability of zirconia. Multiple artificial aging methods, such as thermomechanical cycling, hydrothermal aging, and chemical immersion, are employed to assess their behavior under these conditions accurately. These methods simulate the oral environment to predict the material’s resistance to wear, fracture, and degradation over time. Studies consistently highlight that aging processes can significantly alter the color of zirconia. For instance, hydrothermal aging, which mimics the effects of moisture at elevated temperatures, can induce low-temperature degradation or tetragonal to monoclinic phase transformation. This transformation is accompanied by microcracking, surface roughness, and increased opacity, which may compromise the esthetic quality of restorations. Also, prolonged exposure to acidic conditions has further exacerbated surface changes, potentially affecting translucency and color stability. Mechanical aging through cyclic loading also impacts zirconia’s optical and structural integrity. Fatigue stresses can propagate cracks and induce changes in surface texture, leading to diminished light transmission. Furthermore, combining aging factors, such as simultaneous thermomechanical cycling and pH exposure, amplifies the degradation effects, providing a more comprehensive understanding of zirconia’s long-term performance. These findings underscore the importance of optimizing zirconia’s composition and sintering parameters to enhance resistance against aging-induced changes. Advanced formulations, such as multilayered zirconia with graded translucency and strength, are being developed to address these challenges. Such innovations aim to maintain zirconia’s esthetic and mechanical properties over extended clinical use.

### 2.6. Effect of Zirconia Type on the Color Change in Monolithic Zirconia Ceramics

Zirconia ceramics are optically semi-translucent and structurally based on yttria-stabilized tetragonal zirconia polycrystals. These ceramics are categorized into four types: low, medium, super, and ultra translucency. Moreover, multilayer zirconia ceramics have been developed to replicate a natural tooth’s translucency and color gradient from the incisal to the cervical section [53]. The translucency of zirconia material has been enhanced through various modifications to achieve restorations with properties similar to the natural tooth structure. Multiple types of zirconia are used in dental applications, including partially stabilized zirconia (PSZ), tetragonal zirconia polycrystal (TZP), zirconia toughened alumina (ZTA), and fully cubic stabilized zirconia (CSZ). With the development of new generations of zirconia, significant efforts have been made to enhance the material’s esthetic properties, particularly its color and translucency. These improvements are achieved by modifying the microstructure of zirconia, such as altering the composition of Y-TZP to influence grain size, phase distribution, and light transmission. For instance, higher yttria content in newer generations, like 4Y-PSZ and 5Y-PSZ, increases translucency by reducing the light-scattering tetragonal phase while balancing strength and toughness. These advancements aim to make zirconia restorations more natural-looking and suitable for demanding esthetic zones, bridging the gap between mechanical durability and visual appeal [16,32]. Restorations made with the full-contour technique using 4Y-TZP-MT are the darkest and most translucent, while those made with 3Y-TZP-LT are the lightest and least translucent [17].

Another factor affecting zirconia ceramics’ optical properties is the production technique. Various methods are used in the production of zirconia restorations. Since its introduction in the 1970s, subtractive computer-aided manufacturing technology, such as milling, has become the primary method for producing zirconia restorations. These restorations can be made using soft machining (milling of pre-sintered blocks followed by sintering) and hard machining (milling of fully sintered blocks). The soft machining method, commonly used for yttrium-stabilized zirconia, results in a homogeneous distribution of components and a small pore size. The material undergoes a 25% volumetric shrinkage during sintering, achieving its final mechanical properties. This method produces stable cores with predominantly tetragonal zirconia and minimal monoclinic phase [75]. In contrast, hard machining eliminates shrinkage but may cause phase transformation, leading to low-temperature degradation and microcracks, which can shorten the restoration’s lifespan. Hard machining also requires specialized machines and durable cutting tools. Although hard machining offers better marginal fit than soft machining because it does not require sintering and thus avoids shrinkage, it negatively impacts the mechanical properties of zirconia [76]. On the other hand, additive manufacturing (AM), also known as 3D printing processes, offers a promising alternative to traditional subtractive methods. Three-dimensional printing has become an indispensable tool in biomedical engineering in recent years. This technology enables the creation of three-dimensional structures by depositing materials layer by layer, guided by a digital model. Three-dimensional printing facilitates the production of complex geometric designs with high precision, making it widely applicable in dentistry [77,78]. A systematic review by Alghouli et al. [79] concluded that the esthetic performance of 3D-printed zirconia crowns was superior to milled zirconia crowns. Specifically, 3D-printed zirconia crowns exhibited a significantly better color match and contour alignment with adjacent natural teeth, enhancing their overall appearance and blending seamlessly within the oral cavity. This superior esthetic outcome can be attributed to the precision and customizability offered by 3D printing technology, which allows for finer detail and greater adaptability to individual patient needs. These findings underscore the potential of 3D-printed zirconia crowns as a promising alternative to traditionally milled zirconia restorations. Recent research on 3D-printed zirconia ceramics has increasingly focused on stereolithography-based technologies, which are considered highly promising. A few manufacturers now offer stereolithography-based zirconia printing, showing potential for dental applications [75]. Studies related to 3D-printed zirconia ceramics generally focused on the microstructure of the material, mechanical properties, and dimensional accuracies [80,81,82,83,84]. There is a lack of information on the factors affecting the color of 3D-printed zirconia ceramics.

Due to the complex optical properties of natural teeth, achieving optimum color matching and pleasing esthetics in dental restorations has always been challenging for dentists. Dentists can achieve successful restorations with sufficient knowledge about dental materials’ usage instructions and optical properties [85]. Studies have shown that the type of zirconia block used affects optimum color match and esthetics [85,86,87]. However, there are limited studies in the literature on this subject. According to the differences in yttrium oxide content, different types of zirconia have different translucency values, and the increase in yttrium content increased the translucency [3]. The studies on the effect of zirconia type on the color change in monolithic zirconia ceramics are summarized in Table 6.

These studies have shown that the optical properties of zirconia restorations are influenced by material composition and translucency properties. Studies on monolithic multilayer pre-colored zirconia ceramics with different yttria levels and thicknesses indicate that high-translucency zirconia exhibits more significant color variations, with some types exceeding clinically acceptable color differences. Additionally, investigations into the effects of yttria content and thickness on zirconia’s optical properties suggest that 4Y-PSZ and 5Y-PSZ ceramics result in improved translucency and color accuracy compared to 3Y-TZP. These findings highlight the importance of selecting the appropriate zirconia type and translucency level to achieve optimal esthetic outcomes in zirconia restorations.

## 3. Conclusions

**Figure 2:**
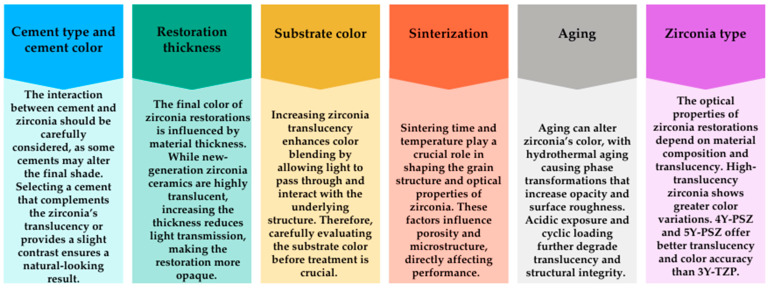
A brief summary of the factors affecting the color change in monolithic zirconia ceramics.

In this narrative review, the factors affecting the color change in monolithic zirconia ceramics are categorized into six main groups: cement type and color, restoration thickness, substrate color, sintering, aging, and zirconia type. A brief summary of these factors is shown in Figure 2.

Several types of zirconia ceramics are available on the market, each with different properties. Studies comparing these various zirconia ceramics have shown that the material type affects the optical properties of restorations. To obtain an esthetic restoration, it is essential to examine existing conditions, such as the color of the abutment tooth and the amount of preparation required, before beginning the treatment process. Therefore, dentists need to understand the properties of various zirconia ceramics to select the most appropriate material according to the patient’s needs. Additionally, the intraoral application of the restoration should be performed using the proper cementation technique. Future research should focus on a more detailed examination of the optical and mechanical properties of different types of zirconia ceramics. Developing new materials and identifying the most suitable restoration methods for clinical applications are expected to play a crucial role in enhancing both the esthetic and functional success of zirconia restorations. Research should focus on developing new material formulations that improve the esthetic and mechanical properties of zirconia ceramics, particularly those that enhance light transmittance and durability. Long-term clinical studies must determine how zirconia restorations perform in the intraoral environment over time. These studies could help assess zirconia ceramics’ durability, color changes, and mechanical stability. Standardizing the tests to evaluate zirconia ceramics’ optical and mechanical properties could improve the comparability of data from different studies. Future research could also focus on several key aspects to enhance the esthetic and functional success of zirconia restorations. First, studies could explore the development of new zirconia formulations aimed at improving esthetic and mechanical properties by discovering additives or processing techniques.

Additionally, more research is needed on the long-term clinical outcomes of zirconia restorations, including their durability, color stability, and functional performance. Comparative studies between zirconia and other restoration materials like porcelain could identify situations where zirconia offers distinct advantages regarding esthetic harmony and longevity. Research into the interaction between zirconia and cement is also crucial, as it would be valuable to understand which cement types achieve the best color match with zirconia and prevent color changes over time. Furthermore, studies on the impact of zirconia veneer thickness on esthetics could help determine the minimum thickness required to ensure color harmony and durability. Finally, there is a need to develop more precise spectrophotometric methods to measure the color match of zirconia restorations. These studies could significantly contribute to achieving more accurate color matching in clinical practice, thus enhancing the overall esthetic success of zirconia restorations.

## Figures and Tables

**Table 1:** Studies on the effects of cement type and cement color on the color change in monolithic zirconia ceramics.

Authors	Aim	Cement Type and/or Color	Study Design	Result
Malkondu et al. [39]	The aim of this study was to evaluate color changes regarding the perceptibility and acceptability of monolithic zirconia-and-cement combinations with two monolithic zirconia thicknesses and three types of cement.	Conventional glass ionomer cement, resin-modified glass ionomer cement, and resin cement from the same manufacturer were applied to the surfaces of the specimens.	Monolithic zirconia material (5Y-PSZ) (Ceramill Zolid; Amann Girrbach, Koblach, Austria) was used in two different thicknesses (0.6 mm, 1 mm) and with three different types of cement: RelyX U200 [3M ESPE, St. Paul, MN, USA] resin cement, RelyX Luting [3M ESPE, USA] resin-modified glass ionomer cement, and Ketac Cem Radiopaque [3M ESPE, USA] conventional glass ionomer cement. Color differences (∆E_ab_) were calculated using the L, a, and b values obtained from the specimens.	Specimen thickness and cement type significantly affected the ∆L, ∆a, ∆b, and ∆E_ab_ values (*p* ˂ 0.05). The smallest color change was observed in the 1 mm thickness group with resin-modified glass ionomer cement (∆E = 2.23), while the greatest color change was observed in the 0.6 mm thickness group with resin cement (∆E = 5.64).
Bayındır et al. [40]	The aim of this in vitro study was to examine the effects of different resin cement colors and material thicknesses on the color and translucency of high-translucency monolithic zirconia.	A transparent and opaque self-etch adhesive resin cements were tested.	Eighty disk-shaped monolithic zirconia specimens (3Y-TZP) (Kuraray Noritake Dental, Okayama, Japan) with a 10 mm diameter were fabricated in four different thicknesses (0.5 mm, 1 mm, 1.5 mm, and 2 mm). The specimens were divided into two subgroups per thickness based on the use of either transparent or opaque self-etch adhesive resin cement (Panavia V5, Kuraray Noritake Dental Inc., Okayama, Japan). Color measurements (CIE L*, a*, and b*) were taken before and after cementation using a spectrophotometer. The translucency parameter (TP) and color change (ΔE_ab_) were then calculated.	Statistical analyses revealed that thickness and cement opacity significantly influenced translucency parameter (TP) and color change values (ΔE_ab_), with thinner specimens exhibiting more excellent translucency and more noticeable color changes due to cement opacity. Monolithic zirconia-clear cement had the lowest value (1.19 for 2 mm), while monolithic zirconia-opaque cement had the highest ΔE value (8.05 for 0.5 mm). These findings highlight the combined effects of material thickness and cement type on the esthetic outcomes of zirconia restorations.
Tabatabaian et al. [41]	The aim of this study was to evaluate the effects of four different cements on the color attributes of a zirconia ceramic.	Four types of cements were used: glass ionomer, Panavia F 2.0, zinc phosphate, and TempBond.	Forty zirconia ceramic disk specimens (Luminesse High Strength 98 mm Discs #5113, Talladium, Valencia, CA, USA) were fabricated and cemented to composite substrates using four different types of cement (glass ionomer, Panavia F 2.0 zinc phosphate, and TempBond). A spectrophotometer measured the specimens’ L, a, and b values before and after cementation. ΔE_ab_ values were compared to the perceptibility threshold of ΔE_ab_ = 3.3.	Cement type affected the color of zirconia ceramic. ΔE_ab_ values of zinc phosphate and Tempbond cement were found to be beyond the perceptibility threshold. However, ΔE_ab_ values of glass ionomer and Panavia F 2.0 cement were within the perceptibility threshold.
Khosravani et al. [42]	The aim of this study was to evaluate the effect of ceramic thickness and resin cement shade on the final color of different layers of ultra-translucent multilayered (UTML) zirconia veneers.	The specimens of each thickness were divided into five groups based on resin cement shade: universal, clear, brown, white, and opaque.	Monolithic zirconia blocks of A1 shade Katana UTML (5Y-PSZ) (Kuraray Noritake Dental, Okayama, Japan) were prepared in 0.7 mm and 0.5 mm thicknesses, with 45 specimens in each group. The specimens were divided into five groups according to the cement color (universal, transparent, brown, white, and opaque). Each cement was applied to the ceramic specimens and the composite substrate, and the color values were recorded before and after the cement application. The color difference for each specimen was calculated using the ΔE_00_ formula.	ΔE_00_ values were significantly influenced by resin cement shade, ceramic thickness, and different layers. In 0.5 and 0.7 mm thick ceramics, the brown shade exhibited the highest ΔE_00_ values (4.49 ± 0.72 and 3.07 ± 0.81, respectively). White and clear shades showed the lowest ΔE_00_ values (0.5 mm: 2.44 ± 1.62, 0.7 mm: 2.22 ± 0.94, respectively).
Sakrana et al. [43]	The aim of the study was to evaluate the effect of resin cement on the color stability of lithium disilicate and zirconia restorations immersed in coffee after aging.	The resin cement types, G-CEM LinkForce (GC Corporation, Tokyo, Japan) and Panavia SA Cement Plus Automix (Kuraray Noritake Dental, Okayama, Japan) were tested.	Eighty maxillary premolars were prepared for lithium disilicate or zirconia restorations (high translucent zirconia), which were bonded with different types of resin cement (G-CEM LinkForce or Panavia SA Cement Plus Automix). All specimens were aged (240,000 loading cycles followed by 10,000 thermal cycles) and then immersed in coffee (for 18 h). The preheating temperature is 25 °C or 54 °C). Color differences were measured using a reflectance spectrophotometer. The ΔE_00_ values were calculated.	Cement type significantly affected the color stability of lithium disilicate and zirconia restorations. A significant difference (*p* = 0.047) was observed between LinkForce (2.28 ± 0.48) and Panavia SA (2.15 ± 0.46) cement. A preheating temperature of 54 °C enhanced the color stability of the zirconia and lithium disilicate.

**Table 2:** Studies on the effect of the restoration thickness on the color change in monolithic zirconia ceramics.

Authors	Aim	Tested Thicknesses	Study Design	Results
Tabatabaian et al. [45]	The aim of the study was to evaluate the effects of veneering porcelain thickness and background shade on the shade match of zirconia-based restorations.	Veneering porcelain thicknesses of 1.6, 1.8, 2.0, and 2.2 mm were tested.	Zirconia blanks 3Y-TZP (VITA YZ T, VITA Zahnfabrik, Bad Säckingen, Germany) of four different thicknesses (1.6, 1.8, 2.0, and 2.2 mm) were placed on three different backgrounds: A2-shaded composite resin, nickel-chromium alloy (NC), and amalgam (AM). The color of the specimens was measured using a spectrophotometer, and the CIELab values were recorded. The color differences ΔE_ab_ between the specimens and the A2 VITA classical shade were calculated. These ΔE_ab_ values were compared to the acceptability threshold of ΔE_ab_ = 3.7.	ΔE_ab_ was influenced by the thickness of the veneering porcelain, the background shade, and their interaction (*p* < 0.05). The minimum veneering porcelain thickness required was 2 mm for NC and 1.8 mm for AM to achieve a proper shade match.
Oh et al. [46]	The aim of the study was to evaluate the effect of abutment shade, ceramic thickness, and coping type on the final shade of zirconia all-ceramic restorations.	Three types of disk-shaped zirconia coping specimens were fabricated and veneered with IPS e.max Press Ceram (shade A2) for 1 mm and 1.5 mm total thicknesses.	Zirconia coping specimens were fabricated from three different materials: Lava (3Y-TZP, 3M ESPE, St. Paul, MN, USA), Cercon (3Y-TZP, (Degudent GmbH, Hanau, Germany)), and Zirkonzahn (3Y-TZP, Zirkonzahn, Gais, Italy). These copings were veneered with IPS e.max Press Ceram (Ivoclar Vivadent, Schaan, Liechtenstein) (A2) to achieve total thicknesses of 1 mm and 1.5 mm. Abutment specimens were made from gold alloy, base metal (nickel-chromium) alloy, and composite resin in four shades (A1, A2, A3, A4). The color difference (ΔE_ab_) was calculated using the CIELab formula.	The abutment shade, ceramic thickness, and coping material significantly influenced the final shade of the zirconia restorations. The highest ∆E value (>2.6) was observed between the gold alloy and A2 resin abutments for the 1 mm-thick zirconia specimens.
Tabatabaian et al. [47]	The aim of this study was to find proper ceramic thickness-cement combinations for color matching of high-translucency monolithic zirconia restorations.	Six different thicknesses of 0.7, 0.9, 1.1, 1.4, 1.6, and 1.8 mm were tested.	Five types of cement were used to bond three hundred A2-shade high-translucency zirconia (5Y-PSZ) disk specimens, with thicknesses of 0.7, 0.9, 1.1, 1.4, 1.6, and 1.8 mm, to A3.5 shade composite resin backgrounds. Color parameters were measured before and after cementation and ΔE_00_ values were calculated to assess the color differences: ∆E_1_ between before and after cementation, ∆E_2_ between the A2 VITA classical shade (target) and specimens before cementation, ∆E_3_ and between the target and specimens after cementation.	Opaque cement and adequate ceramic thicknesses matched the color for high-translucency monolithic zirconia restorations against A3.5-shade backgrounds.
Kang et al. [48]	The aim of this in vitro study was to characterize the effect of thickness on the color accuracy of high-translucency monolithic multilayer pre-colored zirconia.	Four thicknesses were tested: 0.5, 1.0, 1.5, and 2.0 mm.	Plate-shaped, A2 shade high-translucency monolithic multilayer pre-colored zirconia specimens (SHT Multilayer 3Y-TZP, AT Multilayer 5Y-PSZ, and 3D Multilayer 4Y-PSZ + 5Y-PSZ) were tested in 0.5, 1.0, 1.5, and 2 mm thicknesses. The color attributes of these specimens against gray or A2 substrates were measured to assess color accuracy, including color differences (ΔE_00_) compared to the Vita shade guide and chroma.	The thickness influenced the color accuracy of various high-translucency monolithic multilayer pre-colored zirconia types. The optimal thickness for color accuracy was found to be 1.0 mm.

**Table 3:** Studies on the effect of substrate color on the color change in monolithic zirconia ceramics.

Authors	Aim	Substrate Color	Study Design	Results
Comba et al. [29]	The aim of this in vitro study was to evaluate the effects of substrate and cement shades on the translucency and color of lithium-disilicate and zirconia ceramics.	A nanofilled resin-based composite marked Filtek Supreme XTE (3M ESPE, St. Paul, MN, USA) in A2 and A4 shades has been used as the base material.	Two light-cured resin types of cement (RelyX Veneer Cement; 3M, USA; Choice 2 Veneer Cement; Bisco Dental, Schaumburg, IL, USA) with a 0.1 mm thickness were tested. Two monolithic ceramics (3Y-TZP zirconia, lithium disilicate glass-ceramic) and different colored composite substrates were used for 12 combinations. L, a, and b color parameters were measured, and the ΔE_00_ formula was used to calculate the color differences in the study.	Statistically significant effects were found for ceramic material, cement shade, and substrate color (*p* < 0.05). Opacity was notably higher with the use of white opaque cement. The ceramic type and cement shade significantly impacted the L, a, and b values. Therefore, these factors can influence ceramic restorations’ final translucency and color.
Suputtamongkol et al. [49]	The aim of the study was to determine the effect of the background substructure color on the overall color of a zirconia-based all-ceramic crown.	Abutments with metal post and core, abutments with a prefabricated post, and composite core build-up were tested.	Seven premolar and six molar zirconia crowns were cemented onto abutments with metal posts and cores in the first and second groups. Eight molar zirconia crowns were cemented onto abutments in the third group, which included a prefabricated post and composite core build-up. Color measurements of all ceramic crowns were recorded before try-in and before and after cementation. The CIELab (ΔE_ab_) formula was used to calculate the color differences.	No significant differences were found in the L, a, and b values of zirconia crowns cemented onto either metal cast posts and cores or prefabricated posts and composite cores. However, the color of the background substructure can influence the overall color of posterior zirconia restorations, even with clinically recommended core thickness.
Sonza et al. [50]	The aim of the study was to evaluate the influence of the substrate and cement on the final color of ceramic crowns.	Metal (M) and resin (R) were the tested substrate abutments.	Crowns were fabricated using two all-ceramic systems (YZ, 3 mol% yttria-stabilized tetragonal zirconia (3Y-TZP); IZ, alumina-based zirconia-reinforced glass-infiltrated ceramic) and one metal-ceramic system (MC; n = 8). Metal (M) and resin (R) were the substrate abutments. For the resin substrate, crowns were evaluated in both seated (R) and cemented (R-C) conditions. CIELab color coordinates were obtained for the experimental groups, and three different color-difference metrics were used: ΔE*_ab_, ΔE_00_ (1:1:1), and ΔE_00_ (2:1:1).	The substrate and cement affected the final color of zirconia-based ceramic crowns, but the color differences corresponded to an acceptable match.
Tabatabaian et al. [51]	The aim of this study was to evaluate the effect of three different core materials on the masking ability of zirconia ceramic.	A white teflon material (W, white), nickel-chromium alloy (NCA), non-precious gold alloy (NPGA), and zirconia ceramic (ZRC) were tested.	Ten zirconia disk specimens (Luminesse High Strength/Low Translucency, 98 mm Discs #5113; Talladium, Valencia, CA, USA) were fabricated, each 0.5 mm thick and 10 mm in diameter. They were placed on four different substrates: A white (W) substrate (control) and substrates of nickel-chromium alloy (NCA), non-precious gold alloy (NPGA), and ZRC. ΔE values were calculated to assess color differences. Using a spectrophotometer, the study measured the specimens’ L*, a*, and b* values. The color change (ΔE_ab_) values were calculated to determine the color differences between the test and control groups and then compared with the perceptual threshold (ΔE = 2.6).	Statistical analysis showed significant differences between the groups’ L, a, b, and ΔE values. ZRC could not mask the examined core materials well.
Ansarifard et al. [52]	The aim of this study was to examine the impact of substrates, ceramic shades, and brands on the color and masking ability of highly translucent monolithic zirconia (HTMZ).	Non-precious gold alloy (NPG) Nickel–chromium alloy (Ni-Cr) composite resin (shade A2) and zirconia (shade A2) were tested.	One hundred and fifty-six 1 mm-thick highly translucent monolithic zirconia disks in shades A1, A2, and A3 were fabricated from Dental Direkt and Kerox zirconia (3 mol% yttria-stabilized tetragonal zirconia polycrystalline (3Y-TZP) high translucent monolithic zirconia (A1, A2, A3). The disks were placed over four 3 mm-thick substrates (nickel-chromium alloy, non-precious gold alloy, zirconia A2, and resin composite A2), and color measurements were taken with a spectrophotometer. The study analyzed color differences (ΔE) using ΔE_ab_ and ΔE_00_ formulas.	The results showed that substrate type and shade significantly influenced ΔE_ab_ (color change values), while ΔE_00_ (ΔE2000; CIEDE2000 color differences) was additionally affected by the ceramic brand. Most ceramic-substrate groups demonstrated color differences within clinically acceptable and perceptible ranges (clinically perceptible: ΔE_ab_ ≥ 1.3 and ΔE_00_ ≥ 0.8; clinically acceptable: 0.8 < ΔE_00_ ≤ 1.8 and 1.3 < ΔE_ab_ ≤ 2.7). However, the non-precious gold alloy (NPG) substrate exceeded the perceptible range, with ΔE_00_ values from 1.1 ± 0.11 to 1.8 ± 0.31 and ΔE_ab_ values from 1.61 ± 0.15 to 2.16 ± 0.36. A strong correlation (r = 0.974, *p* < 0.001) was observed between ΔE_ab_ and ΔE_00_, indicating that both metrics are reliable for assessing color differences. Variations in ceramic brands and shades caused notable ΔE changes, but the overall color differences remained clinically acceptable.
Ghoveizi et al. [53]	The purpose of this in vitro study was to assess the color match of ultra translucency multilayer zirconia restorations with different designs and backgrounds.	The specimens were seated on five different backgrounds: shade B2 composite resin, shade B2 zirconia, copper-colored metal alloy, silver-colored metal alloy, and the prepared central incisor.	Thirty ultra-translucent multilayer zirconia crowns in VITA classical shade B2 were prepared. The crowns were divided into three groups: veneered zirconia with a trestle design (VZT), veneered zirconia with a dentin core design (VZD), and full-contour zirconia (FCZ). In VZT and VZD groups, zirconia was layered with feldspathic veneering ceramic. The crowns were placed on five backgrounds: Composite resin, zirconia, copper-colored metal, silver-colored metal, and the prepared central incisor. CIELab values of the labial middle sections were measured, and color differences (ΔE*_ab_) were calculated compared to a shade B2 VITA tab, with a clinically acceptable threshold of ΔE*_ab_ = 3.7.	Mean ΔE*_ab_ values ranged from 1.17 to 8.48, with significant effects from restoration design, background type, and their interaction (*p* < 0.001). VZT on all backgrounds and VZD on silver-colored metal showed color mismatches, while VZD on other backgrounds and FCZ on all backgrounds achieved acceptable color matches.

**Table 4:** Studies on the effect of sinterization on the color change in monolithic zirconia ceramics.

Authors	Aim	Sinterization Process	Study Design	Result
Juntavee et al. [55]	The aim of this study was to investigate the effect of sintering temperature and time on the color characteristics of monochrome (Mo) and multilayer (Mu) 5 mol% yttria-partially stabilized zirconia (5Y-PSZ).	The specimens were sintered at three different temperatures: decreasing (Td: 1450 °C), regular (Tr: 1500 °C), and increasing (TI: 1550 °C), combined with four sintering durations: extremely short (He: 10 min), ultrashort (Hu: 15 min), short (Hs: 30 min), and regular (Hr: 135 min).	A total of 300 specimens (dimensions: 10 mm × 20 mm × 2 mm) were prepared from two 5Y-PSZ ceramics, monochromatic and multilayered, with cervical (C), middle (M), and incisal (I) regions represented. These specimens were sintered at three different temperatures combined with four sintering durations. Each group consisted of 15 specimens. The color properties, including whiteness index (EW), translucency parameter (TP), contrast ratio (CR), opalescence parameter (OP), and color difference (ΔE_diff_), were analyzed using the CIELab system.	Significant differences in color parameters were observed based on the zirconia type, sintering temperature, sintering time, and their interactions (*p* < 0.05). Higher sintering temperatures and longer times resulted in larger grain sizes, reduced tetragonal-to-monoclinic phase transformation, and significantly increased translucency parameter (TP) and opalescence parameter (OP). They decreased contrast ratio (CR) and color difference (ΔE_diff_) (*p* < 0.05). Lower sintering temperatures and shorter sintering times led to clinically unacceptable color outcomes. Higher sintering temperatures and longer sintering times are recommended for optimal optical properties.
Ebeid et al. [56]	The aim of this study was to evaluate the effect of different sintering parameters on color reproduction, translucency, and biaxial flexural strength of monolithic zirconia.	The specimens were divided into three groups based on different sintering temperatures (1460 °C, 1530 °C, and 1600 °C). Each group was further divided into three subgroups, which were exposed to sintering holding times of 1, 2, and 4 h.	Translucent zirconia disks (4Y-PSZ) with a diameter of 15 mm, a thickness of 1 mm, and shade A3 were milled and categorized based on different sintering temperatures (1460 °C, 1530 °C, and 1600 °C) into three groups. Each group was further subdivided into three subgroups (n = 10), which were exposed to sintering holding times of 1, 2, and 4 h. The specimens were placed on a neutral gray background, and the CIELab coordinates were measured using a spectrophotometer. ΔE_00_ values below 3.0 were considered “clinically imperceptible”, ΔE_00_ values between 3.0 and 5.0 were considered “clinically acceptable”, and ΔE_00_ values above 5.0 were considered “clinically unacceptable”.	There was no significant difference in the ΔE_00_ value between the 2 h and 4 h sintering times, while both times showed a significant decrease compared to the 1 h sintering time. All subgroups showed acceptable color results, and the 1600 °C sintering temperature provided clinically indistinguishable color outcomes.
Salah et al. [59]	The aim of this in vitro study was to investigate the effect of different rapid sintering protocols on the color and translucency of cubic and tetragonal zirconia ceramics.	Each zirconia type was divided into three groups based on sintering protocols: conventional (control), speed, and super speed.	Sixty disk-shaped zirconia specimens (1 mm thick) were made from cubic (DD CubeX2) (5Y-PSZ) and tetragonal (DD Bio ZX2) zirconia (5Y-PSZ). Each zirconia type was divided into three groups based on sintering protocols. The translucency of each group was evaluated using the translucency parameter and contrast ratio, with color differences calculated relative to the conventional sintering group. The ΔE_ab_ threshold values were set at 1.2 for perceptibility and 2.7 for acceptability.	Speed and superspeed sintering protocols significantly reduced the translucency of cubic and tetragonal zirconia (*p* < 0.001), with superspeed sintering causing more significant color changes than speed sintering (*p* < 0.001). When speed sintering was performed, the color difference between cubic (ΔE_ab_ = 1.44) and tetragonal (ΔE_ab_ = 1.61) zirconia was acceptable compared to their conventionally sintered counterparts. However, with super-speed sintering, the ΔE_ab_ for cubic and tetragonal zirconia was measured as 17.79 and 5.6, respectively, indicating a perceivable and unacceptable color change. The study concluded that rapid sintering protocols substantially affected the color and translucency of these zirconia types.
Yang et al. [60]	The aim of this study was to investigate the changes in the translucency and color of four different multilayered zirconia materials when the sintering temperature were inaccurate.	Each zirconia type was divided into five subgroups based on sintering temperature: L1 (5% lower), L2 (2.5% lower), R (recommended), H2 (2.5% higher), and H1 (5% higher).	Two hundred zirconia specimens (11 × 11 × 1.0 mm) were prepared from four multilayered zirconia types (5Y-PSZ): Upcera TT-GT (UG), Upcera TT-ML (UM), Cercon xt ML (CX), and Lava Esthetic (LE), and were divided into five subgroups based on sintering temperature. After sintering, color coordinates were measured, and translucency parameter (TP) values and color differences (ΔE) between non-recommended and recommended sintering temperatures were calculated. Mean ΔE_00_ values below 1.25 were assumed “clinically imperceptible”, while mean ΔE_00_ values above 2.23 were assumed “clinically unacceptable”.	Sintering temperature deviations significantly impacted multilayered zirconia ceramics’ translucency (TP) and color (ΔE_00_). TP values varied with temperature, except in the cervical and body sections of UG, with higher temperatures increasing TP for CX but decreasing it for LE. ΔE_00_ values were clinically imperceptible for UM and CX at the most inaccurate temperatures and clinically acceptable for all materials (ΔE_00_ < 2.23).
Giti et al. [61]	The aim of this study was to evaluate the effect of sintering temperature on color stability and translucency in zirconia systems with low, high, and ultra-high translucencies.	Each group was divided into subgroups sintered at 1450 °C or 1550 °C.	Sixty zirconia disks: DD Bio ZW (3Y-TZP), DD Bio ZX2 98 (3Y-TZP), and DD CubeX2 (5Y-PSZ) with low, high, and ultra-high translucencies were prepared, and each group was divided into subgroups sintered at 1450 °C or 1550 °C. Baseline color and translucency parameters were recorded, followed by 30 days of coffee immersion, after which measurements were repeated. Color changes (ΔE_ab_) and translucency changes (ΔTP) were calculated using the CIELAB formula.	Results showed significant differences in ΔE among zirconia translucencies (*p* < 0.001) but no differences between sintering temperatures (*p* = 0.712). Low-translucency zirconia had higher ΔE values than high- and ultra-high-translucency groups (*p* < 0.05), which were not significantly different. Sintering temperature did not impact color or translucency changes after coffee immersion. Higher-translucency zirconia showed better color stability.
Hassan Shohdy et al. [62]	The aim of this study was to investigate the influence of speed sintering on the microstructure and optical properties of ultra-translucent multilayered cubic zirconia.	The specimens were divided into two groups based on the conventional (control) and speed sintering techniques. The conventional sintering was performed at 1550 °C for 2 h, while the speed sintering was performed at 1560 °C for 0.5 h.	Ultra-translucent cubic zirconia 5Y-PSZ (Katana UTML; Kuraray Noritake Dental Inc., Aichi, Japan) was sectioned into 80 specimens, with 20 specimens from each blank layer. The specimens were categorized into two groups: speed sintering and conventional sintering. Measurements of translucency, opalescence, chromaticity, and color differences were obtained using a spectrophotometer. The study recorded L*, a*, and b* color values, and the color software application (Cary Win UV; Agilent Technologies, Santa Clara, CA, USA) was used for this process. TP, OP, C*ab, and ΔEab values were calculated.	Results indicated that speed sintering significantly reduced translucency and opalescence, except the dentin layer’s opalescence. Chromaticity decreased in less chromatic layers and increased in more chromatic layers with speed sintering. The mean color change ranged from 0.65 to 1.25 across different layers, and crystal size decreased with speed sintering. However, no clinically noticeable color change was observed compared to conventional sintering.
Engler et al. [63]	The aim of this in vitro study was to evaluate the color and translucency of zirconia subjected to different sintering temperatures and aging.	All specimens were divided according to the sintering temperature: 1400 °C, 1450 °C, and 1500 °C.	One-hundred-and-eighty disk-shaped (1.5 mm-thick) specimens were produced from translucent zirconia blocks: 5Y-TZP (DD cubeX2 white, DD cubeX2 A2 shade), 3Y-TZP (DD Bio ZX2 white), and 3Y-TZP (Lava Plus white). All disks were divided according to the sintering temperature: 1400 °C, 1450 °C, and 1500 °C. The specimens were subjected to an aging protocol in an autoclave for 5 h and 20 h. ΔE_00_, ∆L′, ∆C′, ∆H′, and TP were calculated using CIEDE2000, and CR was obtained using CIEXYZ.	The experimental sintering temperatures (1400 °C and 1500 °C) promoted color changes in almost all the materials evaluated. The most significant ∆E_00_ was found at 1400 °C, with the most remarkable difference in the Lava Plus A2 group (5.25), followed by the DD Bio ZX2 White group (4.80). Opacity and saturation values were increased at 1400 °C.
Aly et al. [64]	The aim of this study was to evaluate the effect of different sintering techniques on the color parameters of zirconia ceramic restorations.	Zirconia specimens were divided into three groups: microwave sintering (MS), speed sintering (SS), and conventional sintering (CS). MS involved heating to 1500 °C for 2 h, SS to 1550 °C for 30 min, and CS to 1500 °C for 2 h.	Thirty Zenostar Translucent zirconia (3Y-TZP), shade T2 specimens were prepared (10 mm × 2 mm, thickness). Three groups were created. The color difference (ΔE_ab_) was calculated by using a spectrophotometer.	The sintering process affected the optical and color parameters of zirconia specimens. When comparing each group specimen to known shade tabs (A1, A2, B1, B2, C1), the lowest mean ∆E_ab_ value was recorded for MS (2.50 ± 0.31) when compared with shade tab A1; however, there was no significant difference when compared with group CS (2.75 ± 0.50). The highest ∆E_ab_ (3.72 ± 0.75) was found between SS and MS. However, this difference is considered within the clinically acceptable range (∆E_ab_ 3–5).

**Table 5:** Studies on the effect of aging on the color change in monolithic zirconia ceramics.

Authors	Aim	Aging Process	Study Design	Result
Ângela Mazıero Volpato et al. [68]	The aim of this study was to evaluate the influence of different aging times on the color stability of zirconia that is either veneered or not by ceramic.	All specimens underwent the same accelerated aging protocol. They were aged in an autoclave at 134 °C for 1 h (T2), 2 h (T3), and 4 h (T4), with all procedures performed consecutively on the same day.	Fifteen zirconia disks (3Y-TZP) were fabricated (12.0 mm diameter and 1.0 mm thick) and divided into three groups (n = 5). Group 1 did not receive a veneering ceramic layer; group 2 veneered with a 1.0 mm ceramic layer, and group 3 veneered with a 1.5 mm ceramic layer. Color differences in specimens were calculated after 1 h, 2 h, and 4 h of aging. Color differences (ΔE_00_), lightness (ΔL_0_), chroma (ΔC_0_), and hue (ΔH_0_) were calculated using the CIEDE2000 color difference formula among standard averages, aging times, and tested thicknesses.	In the G1 group, statistically significant differences were observed in lightness (ΔL_0_), chroma (ΔC_0_), and hue (ΔH_0_) (*p* = 0.0001), with a particularly notable increase in chroma difference (ΔC_0_). Distinct color changes (ΔE_00_) = 0.95 were recorded after 4 h of aging. In conclusion, after prolonged aging, discrete color changes can occur in zirconia, mainly when it is in contact with the oral environment, as in abutments and monolithic crowns.
Kim et al. [70]	The aim of this in vitro study was to evaluate the effect of hydrothermal aging on the optical properties, phase transformation, and surface topography of pre-colored monolithic zirconia ceramics.	The specimens were artificially aged in an autoclave at 134 °C under 0.2 MPa for 0, 1, 3, 5, or 10 h (n = 10).	Pre-colored monolithic zirconia specimens (3Y-TZP, 4Y-PSZ) (17 × 17 × 1.5 mm, n = 50) and lithium disilicate glass-ceramic specimens (16 × 16 × 1.5 mm, n = 50) were artificially aged in an autoclave at 134 °C under 0.2 MPa for 0, 1, 3, 5, or 10 h. CIELab color parameters were obtained from spectral measurements. The translucency parameter (TP) and CIEDE2000 color differences (ΔE_00_) were calculated.	Significant interactions were found between aging time and ceramic material on L, a, b values and TP (*p* < 0.001), with the most critical effect on b*. TP values increased for Katana (*p* < 0.014) and in e.max as a function of aging time (*p* < 0.001). TP increased after aging for zirconia (*p* < 0.014) except at 10 h (*p* = 0.389) and for lithium disilicate (*p* < 0.001). TP increased after aging for zirconia (*p* < 0.014) except at 10 h (*p* = 0.389) and for lithium disilicate (*p* < 0.001). Hydrothermal aging affected the optical properties and microstructures of pre-colored monolithic zirconia ceramics, and translucency increased slightly with aging time. In ΔE_00_ analysis, Katana exhibited clinically unacceptable color changes compared to baseline (2.03–2.52 ΔE_00_), while e.max demonstrated stable color matching (0.07–0.23 ΔE_00_).
Miura et al. [71]	The aim of this study was to investigate the effects of low-temperature degradation (LTD) on the L*, a*, and b* values of highly translucent zirconia crowns.	In an autoclave, LTD treatment was applied for 5 h at 134 °C and 0.2 MPa.	Four types of zirconia disks with varying yttria contents IPS e.max ZirCAD LT (3Y-TZP), IPS e.max ZirCAD MT (4Y-PSZ), IPS e.max ZirCAD MT Multi (4Y-PSZ, 5Y-PSZ), IPS e.max ZirCAD Prime (3Y-TZP, 5Y-PSZ) and two shades (A2 and BL) were used to manufacture crowns. The color difference (ΔE_00_) before and after LTD was calculated from each area’s L*, a*, and b* values using the CIEDE2000 formula. Color measurements were taken before and after LTD treatment. The color difference (ΔE_00_) was calculated.	L*, a*, b*, and ΔE_00_ values of four zirconia types were influenced by LTD treatment. Highly translucent zirconia crowns with varying yttria contents showed more significant changes in a* and b* values than in L* after LTD, regardless of shade. Multi2 crowns with 3 and 5 mol% yttria exhibited noticeable color changes due to LTD treatment.
Arindham et al. [72]	The aim of this study was to evaluate the effects of immersion in alcoholic beverages on the surface roughness and color stability of two types of milled zirconia.	The specimens were sintered at 1500 °C for eight hours and then immersed in artificial saliva, red wine, and whiskey three times daily for 30 days.	Sixty cuboid-shaped specimens from two types of milled 3Y-TZP zirconia. (Z1: NexxZr T, Sagemax^®^, Ivoclar Vivadent, Amherst, NY, USA, and Z2: and Cercon^®^ht, Dentsply Sirona, Charlotte, NC, USA) were sintered at 1500 °C for eight hours and immersed in artificial saliva, red wine, and whiskey three times daily for 30 days. Surface roughness and color changes were measured using AFM and a spectrophotometer. Color stability (ΔE) values were measured using the CIE L*a* b system.	Results showed that whiskey caused the highest surface roughness increase in Z1 (137.09 nm) and Z2 (86.15 nm), while red wine led to the most discoloration in both Z1 (2.41) and Z2 (1.94). Whiskey, red wine, and artificial saliva increased surface roughness with no significant differences in color changes.
Abounassif et al. [73]	The aim of this study was to explore the impact of zirconia types, coloring methods, and surface finishing on the color stability of monolithic multilayered polychromatic zirconia after artificial aging, including thermocycling and simulated toothbrushing.	The specimens were subjected to 5000 cycles at 5 °C and 55 °C for 15 s each for thermal cycling. In the tooth brushing simulation, 5000 cycles were performed using a soft toothbrush and a cleaning slurry prepared from a specific mixture.	Eighty square-shaped zirconia specimens were categorized into two types (M3Y-TZP and M6Y-PSZ), with further divisions based on coloring methods (pre-colored and extrinsically colored) and surface finishing techniques (mechanical polishing or glazing). Color stability was evaluated using the CIEDE2000 formula, with thermocycling and toothbrushing simulating artificial aging. Spectrophotometer measurements determined post-aging color changes (ΔE_00_). A value of 0.8 was set for the 50:50% PT, and 1.8 was set for the 50:50% AT to compare changes in ΔE_00_.	The study found significant differences in color stability based on zirconia type, coloring method, and surface finishing. M6Y groups showed more substantial color changes (6.61 ± 1.63) compared to M3Y groups (3.40 ± 2.24), with extrinsically colored specimens exhibiting higher ΔE_00_ when mechanically polished (*p* = 0.004). Surface finishing did not significantly affect ΔE_00_ in pre-colored specimens (*p* = 1.000). The extrinsic coloring paired with glazing is recommended for maintaining color stability in multilayered zirconia restorations, while for pre-colored zirconia, both glazing and mechanical polishing are effective.
Park et al. [74]	The purpose of this in vitro study was to analyze the changes in color, translucency, and hardness of a single layer of multilayered zirconia after an LTD treatment.	Lava Esthetic Zirconia containing 5 mol% yttria (5Y-PSZ) was tested.	Ninety 10 × 10 × 1.0 mm multilayered zirconia specimens (3M Lava Esthetic) were prepared and divided into six groups (n = 15) based on the LTD (no treatment, LTD), and the inherent layer type (incisal, transition, and body) (CIELab) parameters were calculated by spectral reflectance. Color differences (ΔE_ab_) and TP values were measured.	Optical properties were not affected by LTD treatment. Increasing red and yellow and decreasing brightness from the incisal layer to the body layer were observed.

**Table 6:** Studies on the effect of zirconia type on the color change in monolithic zirconia ceramics.

Authors	Aim	Type of Zirconia	Study Design	Result
Vafaei et al. [85]	The aim of this study was to compare the optical properties of Zolid FX, Katana UTML and lithium disilicate laminate veneers.	5Y-TZP (Katana UTML, Kuraray Noritake Dental Inc., Miyoshi, Japan and Zolid FX, (Amann Girrbach AG, Koblach, Austria) was tested.	The maxillary left lateral incisor of a phantom received a laminate veneer preparation. Ten dies were fabricated from a composite resin with A1, A2, and A3 shades. Laminate veneers were fabricated using an A1 shade of Katana UTML, Zolid FX, and IPS e.max CAD ceramics and placed on composite abutments using bleach and white colors of trial insertion paste (TIP). The optical properties of the specimens were measured at the incisal, middle, and cervical thirds. The mean values of L*, a*, and b* color parameters (ΔE_ab_) were calculated based on the laminate material, TIP color, and composite abutment shade in the cervical, middle, and incisal thirds.	The effect of laminate material on the color parameters was significant in all areas (*p* < 0.001) except for the L parameter in the middle and cervical thirds. The composite abutment shade, TIP color, and laminate material should be carefully selected for optimal laminate veneers’ esthetics.
Kang et al. [86]	The aim of this in vitro study was to determine the color accuracy of different types of monolithic multilayer pre-colored zirconia ceramics of varying thicknesses.	3Y-TZP + 5Y-PSZ, 5Y-PSZ, 4Y-PSZ + 5Y-PSZ, and 4Y-PSZ + 5Y-PSZ ceramics were tested.	Eighty specimens with A2 shade, two different thicknesses (1.0 mm and 1.5 mm), and four different zirconia brands (UPCERA EXPLORE [UPEX], KATANA Zirconia STML [STML], Enamel ZR Multi-5 [EZM5], and Aidite 3D Pro Zir [A3DM]) were fabricated and measured using a spectrophotometer against gray, transparent, and A2 backgrounds. The color difference (ΔE*_ab_), translucency parameter (TP), and chroma (C) values for each group were calculated.	The (ΔE*_ab_) values for UPEX and STML zirconia brands exceeded the clinically acceptable threshold values for gray and transparent backing substrates (ΔE > 3.7). In contrast, A3DM zirconia had clinically acceptable (ΔE*_ab_) values for all backing substrates. Color accuracy was mainly affected by the monolithic multilayer pre-colored zirconia ceramic type, and the high transparency of ceramics caused color differences.
Kang et al. [87]	The aim of this study was to investigate variations in yttria levels and thicknesses that affected multilayer monolithic zirconia’s optical properties and fracture loads.	Two types of multilayer monolithic zirconia in the Vita A1 shade were used, each composed of different yttria levels: one was uniformly composed of 3Y with 4.5 to 5.5 wt.% of Y_2_O_3_ (Superfectzir; Aidite Technology, Qinhuangdao, China; denoted as SZ), and the other was a combination of 4Y and 5Y with 4.0 to 10.0 wt.% of Y_2_O_3_ (Aizir; Aidite Technology; denoted as AZ).	Specimens in the Vita A1 shade were tested using 3Y-TZP (SZ) and a combination of 4Y- and 5Y-PSZ (AZ). Optical properties, including color difference (ΔE_WS_) and translucency parameter (TP_00_), were evaluated with a digital colorimeter. For testing optical properties, 15 multilayer monolithic zirconia plate specimens (10 mm × 10 mm) were prepared in four thicknesses: 0.5, 1.0, 1.5, and 2.0 mm. To confirm color accuracy, ∆E_WS_ was calculated based on the color attributes measured on a white substrate.	For ΔE_WS_ values, SZ ranged from 3.6 to 4.0, while AZ exhibited a ΔE_WS_ of 3.9 at 0.5 mm and less than 2.6 at greater thicknesses. TP_00_ values decreased as thickness increased, with AZ generally demonstrating higher translucency than SZ. Multilayer monolithic zirconia with combined yttria levels of 4Y + 5Y (AZ) demonstrated high translucency and accurate color matching.

## Data Availability

The datasets used and analyzed during the present study are available from the corresponding author upon reasonable request.

## Abbreviations

The following abbreviations are used in this manuscript:

Y-TZP	Yttria-stabilized tetragonal zirconia polycrystals
Y-PSZ	Yittria-partially stabilized zirconia
wt.	weight
CAD-CAM	Computer-aided design-Computer-aided manufacturing
TP	Translucency parameter
UTML	Ultra translucent multi-layered
LTD	Low-temperature degradation
